# Dangerous Liaisons: Circulating Tumor Cells (CTCs) and Cancer-Associated Fibroblasts (CAFs)

**DOI:** 10.3390/cancers12102861

**Published:** 2020-10-05

**Authors:** Pablo Hurtado, Inés Martínez-Pena, Roberto Piñeiro

**Affiliations:** 1 Roche-Chus Joint Unit, Translational Medical Oncology Group, Oncomet, Health Research Institute of Santiago de Compostela, Travesía da Choupana s/n, 15706 Santiago de Compostela, Spain; pablo.hurtado.blanco@rai.usc.es (P.H.); ines.martinez.pena@rai.usc.es (I.M.-P.); 2 CIBERONC, Centro de Investigación Biomédica en Red Cáncer, 28029 Madrid, Spain

**Keywords:** circulating tumor cells (CTCs), CTC clusters, cancer-associated fibroblasts (CAFs), metastasis, liquid biopsy

## Abstract

**Simple Summary:**

Circulating tumor cell clusters (CTC clusters) seem to play a primary role in the metastatic spread of cancer, the main cause of death associated with this disease. The metastatic potential of CTCs can be enhanced by the presence within the clusters of cancer-associated fibroblast (CAFs), known to promote cancer invasion and dissemination. In this review, the authors summarize the role of CTC clusters and CAFs on the metastatic process and the current knowledge about the contribution of CAFs to the genesis and metastasis initiating ability of CTC clusters. In addition, they discuss the potential of therapeutically interfering with the CAFs within CTC clusters, as a strategy to reduce their metastatic competency. Lastly, the authors highlight some relevant questions about the biology of these clusters that need to be answered in order to fully understand and be able to limit their contribution to metastasis.

**Abstract:**

The crosstalk between cancer cells and the tumor microenvironment (TME) is a key determinant of cancer metastasis. Cancer-associated fibroblasts (CAFs), one of the main cellular components of TME, promote cancer cell invasion and dissemination through mechanisms including cell-cell interactions and the paracrine secretion of growth factors, cytokines and chemokines. During metastasis, circulating tumor cells (CTCs) are shed from the primary tumor to the bloodstream, where they can be detected as single cells or clusters. The current knowledge about the biology of CTC clusters positions them as key actors in metastasis formation. It also indicates that CTCs do not act alone and that they may be aided by stromal and immune cells, which seem to shape their metastatic potential. Among these cells, CAFs are found associated with CTCs in heterotypic CTC clusters, and their presence seems to increase their metastatic efficiency. In this review, we summarize the current knowledge on the role that CAFs play on metastasis and we discuss their implication on the biogenesis, metastasis-initiating capacity of CTC clusters, and clinical implications. Moreover, we speculate about possible therapeutic strategies aimed to limit the metastatic potential of CTC clusters involving the targeting of CAFs as well as their difficulties and limitations.

## 1. Introduction—Metastasis and the Tumor Microenvironment

Metastasis is a complex process involving different steps and environments [1,2,3]. During this process, genetically unstable tumor cells undergo structural and functional changes that, within a permissive microenvironment, allow them to metastasize to distant organs and tissues [4]. Over the last decades, it has become clear that tumor progression does not exclusively depend on cancer cell-autonomous functions and that tumor stroma reactivity is a key factor. Along disease progression, cancer cells are supported by a dynamic bidirectional crosstalk with the tumor microenvironment (TME) that directly influences disease initiation, progression, organ-specific metastasis, and patient prognosis [5,6]. The TME is composed of cells from mesenchymal (fibroblasts), endothelial (endothelial cells and pericytes), and hematopoietic (immune cells) origins, and the extracellular matrix (ECM) components. 

The role that cells from the stroma such as cancer-associated fibroblasts (CAFs) and tumor-associated macrophages (TAMs), and the ECM play during the earlier steps of metastasis is being unraveled [7,8]. Cancer cells can be assisted by stromal cells in acquiring an invasive phenotype, driven by the genetic program known as epithelial-to-mesenchymal transition (EMT). EMT allows tumor cells to separate from neighboring epithelial cell-cell contacts and acquire a mobile/invasive phenotype, although evidence shows that it is not absolutely required for the release of cancer cells into the bloodstream, or at least it is not a complete EMT process, a.k.a. epithelial–mesenchymal plasticity (EMP) [9]. Once cancer cells are able to invade the surrounding tissue, two major rate-limiting steps in the metastatic cascade are the intravasation and survival in circulation as circulating tumor cells (CTCs) [10,11]. The vast majority of tumor cells in the bloodstream are destroyed by shear stress forces, anoikis due to the detachment of the tumor cells from the extracellular matrix, and immune attack [11,12,13]. In addition, for those cells which survive the transit in circulation, the slow rate of extravasation and proliferation in the stroma at a secondary site is another important limiting step [14]. Therefore, and in spite of the large number of tumor cells that are shed daily into circulation, the metastasis is a very inefficient process [15], as it has been shown by experimental studies [16,17]. Only those cancer cells with the capability to survive in the bloodstream, adapt to the distant tissue and new microenvironment, and induce angiogenesis, will successfully seed metastases. 

## 2. Circulating Tumor Cells (CTCs)

CTCs are cancer cells shed from the primary tumor or metastatic lesions found in the peripheral blood of patients with cancer. The hematogenous spread of cancer cells was reported in the 19th century [18,19]. However, the isolation and characterization of CTCs have been hampered mainly by the combination of two factors, their low frequency in the blood of cancer patients, and until recently, the limited access to a technology capable of enriching few CTCs from a large background of blood cells. In recent years, many different assays were developed for the identification, enumeration, and characterization of CTCs [20]. CTC-enrichment technologies are broadly classified in two groups. On one side, technologies taking advantage of the biological properties of the CTCs, usually the recognition of epithelial cell surface markers, and on the other, taking advantage of physical properties, such as size and density [21]. A relevant example of the first group is the widely used CellSearch^®^ (Menarini Silicon Biosystems, Bologna, Italy) system, based on the immunomagnetic capture of epithelial cell adhesion molecule (EpCAM) expressing cells and their identification as CTCs by positive immunostaining for cytokeratins 8/18/19, and negative for CD45, a white blood cell marker. However, the plasticity on the expression of epithelial and mesenchymal phenotypes observed in CTCs has an impact on the capture efficiency of technologies based on the recognition of epithelial markers such as EpCAM. A growing number of newer technologies combine both strategies. Particularly relevant are the technologies based on microfluidics, which allow a continuous sample processing to reduce target cell loss, and easy integration of various functions (“do everything -on-a-chip”) [22].

CTCs are clinically relevant, indeed, high CTC levels in the bloodstream of prostate, breast, colorectal, lung, and bladder cancer patients are associated with poor prognosis and an increased probability of metastatic disease [23]. For this reason, CTCs isolated from peripheral blood are key for the study of the mechanism of cancer metastasis, and they serve as a source of tumor material for a real time monitoring and prediction of the tumor progression and therapeutic response through a minimally invasive “liquid biopsy”. CTCs can be found in the blood as single cells or as clusters of cells (CTC clusters), although at different frequencies. It is estimated that CTC clusters comprise a 1–30% of the total number of CTCs found in the blood of cancer patients and mouse models [24].

### 2.1. CTC Clusters

By definition, a cluster of CTCs is a group of two or more tumor cells detected in the blood of a cancer patient. A key feature of these groups of cells is the persistent presence of strong cell-cell junctions within the cells of the cluster [25,26]. The existence of CTC clusters was already predicted by Rudolf Virchow, in 1858 [27]. Almost a century later some studies emerged acknowledging the role of CTC clusters in metastasis [28,29]. It was in the 90´s that first studies isolated CTC clusters from the blood of patients with certain types of cancer, such as prostate, colorectal, breast, lung and clear cell renal cell and hepatocellular carcinoma [30,31,32,33,34,35,36]. According to cancer animal models, the proportion of CTC clusters in the blood increases during cancer progression [37]. Importantly, the presence of CTC clusters in the peripheral blood of cancer patients is associated with a worse outcome and an earlier onset of metastatic disease, and despite the need of further confirmation by larger clinical trials, they seem to stand alone as an independent prognostic factor [26,30,31,38,39,40,41,42,43,44,45]. The size of the clusters can vary from 2 up to >100 cells [5] and it correlates with the clinical outcome of patients with metastatic breast cancer [44,45]. 

### 2.2. CTC Clusters Biology and Metastatic Potential

The identification and characterization of CTC clusters have been limited by the technologies applied to their capture and isolation, primarily designed for the isolation of single CTCs. An inadequate technology translates into a low recovery efficiency, inability to separate CTC clusters from single CTCs, and cluster damage and break up during the separation, masking the real number of CTC clusters in the bloodstream [46,47]. However, the recent development of more suitable isolation technologies together with molecular and functional assays with resolution at the single cell level have allowed to obtain some insights into their biology. CTC clusters appear to have a higher potential for metastasis compared to individual CTCs [26]. This metastasis-initiating ability is the consequence of the combination of key biological features derived from the clustering of cancer cells. Thus, specific gene expression profiles seem to be responsible for the hybrid epithelial/mesenchymal phenotype observed in CTC clusters [48] while maintaining the expression of cell-cell adhesion molecules [25,26], an important mediator of pro-survival signals. Moreover, gene expression analyses show the downregulation of apoptosis, immune response, MHC class II antigen presentation, and T-cell activation associated genes [25,49], and the upregulation of proliferation and stemness-associated genes [49]. The “stemness” of CTC clusters seems to be a key contributing factor to their metastatic initiating properties. DNA methylation analyses show that binding sites for stemness-related transcription factors involved in the regulation of proliferation and pluripotency, such as OCT4, NANOG, SOX2 and SIN3A, are hypomethylated in CTC clusters [50]. Moreover, CTC clusters express increased levels of the stem cell marker CD44, which is involved in CTC clustering and metastasis formation [51,52]. Therefore, these characteristics reflect the enhanced metastatic potential of CTC clusters and portray them as main actors in the process of dissemination of carcinomas. In addition, CTC clusters can initiate the formation of polyclonal metastases [25,26,51,53], suggesting the existence of a mechanism of cooperation between cancer cell clones within CTC clusters that can have relevant implications in the disease evolution, and in particular in the diagnosis and treatment of the metastatic disease. For a deeper insight about the biology of CTC clusters, the most recent knowledge is summarized in references [24,46,54].

The isolation and characterization of CTC clusters of cancer patients and animal models have revealed that they can be exclusively formed by tumor cells, known as homotypic CTC clusters, or they can also travel accompanied by non-tumor cells, known as heterotypic CTC clusters. These non-malignant cells are mainly stroma-derived cells, such as fibroblasts and endothelial cells, platelets, and immune cells, such as white blood cells and neutrophils [55]. The close interaction of CTCs with these microenvironmental cells seems to shape the metastatic potential of CTC clusters by enhancing the metastasis-seeding ability of CTCs [48,56,57,58]. Indeed, recent studies show the key role of neutrophils and polymorphonuclear-myeloid-derived suppressor cells (PMN-MDSCs) on enhancing the metastatic potential of CTCs from breast cancer and melanoma [57,58]. A similar role has been suggested for CAFs travelling in the bloodstream within heterotypic CTC clusters [56,59], which will be later reviewed in more detail. 

## 3. Cancer-Associated Fibroblasts (CAFs)

CAFs are one of the most dominant cellular components in the TME and their abundance correlates with worse prognosis in solid cancers [60]. These cells are found in highly aberrant numbers and are different from normal fibroblasts [6]. They seem to be found locked in a permanent activated stage, believed to be mainly due to stromal factor-induced epigenetic changes and not to the accumulation of somatic mutations [61,62]. CAFs are spindle-shaped cells that have important roles in ECM structure remodeling by depositing ECM components (collagens, hyaluronan, fibronectins and laminins) and synthesizing elevated levels of ECM-degrading proteases, promoting tumor fibrosis [63]. ECM structure remodeling by CAFs allows tumor cells to invade through the stroma, and CAFs secretion of growth factors, cytokines and chemokines enables the communication between cancer cells and stromal cells [64]. But CAFs are also involved in a large plethora of processes related to cancer such as tumorigenesis, angiogenesis, metastasis, immunosuppression, drug resistance, maintenance of cancer stemness, and metabolic reprogramming [65].

### 3.1. CAF Origin and Heterogeneity

CAFs are a heterogeneous population of cells, whose origin can be very diverse, with many potential cellular precursors. Nevertheless, it is generally agreed that most CAF derive from local tissue-resident fibroblasts or tissue-specific myofibroblast progenitors that become activated by neighboring tumor cells, TME stimuli, and infiltrating immune cells [66,67,68,69]. However, other cellular precursors of CAFs are fibrocytes recruited from bone marrow and bone marrow-derived mesenchymal stem cells (BM-MSCs) [65,70]. Moreover, the transdifferentiation of epithelial or endothelial cells [71,72], and in a lower proportion adipocytes, pericytes, and smooth muscle cells into CAFs, can be other relevant sources [73,74,75]. All these cellular precursors contribute to the high heterogeneity observed across the diverse CAF subpopulations, both at the functional and gene expression level. Current knowledge in the field supports the existence of specialized and reversible subpopulations of CAFs responsible for the plethora of functions attributed to them, characterized by a considerable plasticity allowing them to switch from one functional state to another in response to both genetic and non-genetic factors [69]. Thus, CAF heterogeneity is evidenced by the variety of biological markers with selective expression patterns that condition their function. Among these markers, the most commonly used to define a CAF are α-smooth muscle actin (α-SMA), fibroblast activation protein (FAP), S100A4 (also known as fibroblast specific protein 1 or FSP1), platelet-derived growth factor receptor-α (PDGFRα) and PDGFRβ [63]. Importantly, the expression of these markers in the tumor stroma is linked to poor outcome in many cancer types [76,77]. The identification of CAFs, based on the study of the expression of these markers in stromal cells, is primarily assessed by antibody-based technologies, such as immunohistochemistry, immunofluorescence, and flow cytometry. It is important to notice that their expression is not restricted to CAFs, and no single marker is expressed in all CAFs, which represents an important limitation for CAF identification and classification. An example of this is the work published by Cremasco et al. They show that both stromal fibroblasts and pericytes of breast tumors express FAP and α-SMA, however, they have different functions, with CAFs mediating ECM remodeling and immunosuppression [78]. Pelon et al. present a recent example of CAF heterogeneity and its impact on CAF function [79]. They identify the existence of four different CAF subpopulations in metastatic lymph nodes (LN) from breast cancer patients based on the combined analysis of five markers by multicolor flow cytometry. Interestingly, the two most abundant subpopulations in LN, and primary tumors, correlate with cancer cell invasion and promote the metastasis of cancer cells through different molecular mechanisms. Moreover, FAP expressing CAFs derived from the different breast cancer subtypes present a differential gene expression profile [80].

### 3.2. Support Role of CAFs in Metastasis

CAFs may play a relevant role in metastasis. They are important mediators of the dissemination of cancer cells from the primary site and the secondary tumor growth at the metastatic site. A clear evidence is reported by Grum-Schwensen et al., who show that tumors developing in mice lacking the S100A4 gene (which lack migrating fibroblasts) never metastasize. However, these tumors acquire metastatic competency when fibroblasts expressing S100A4 are supplied [81]. At primary sites, CAF-derived extracellular matrix proteins affect ECM stiffness, a major determinant of mechanosignaling promoting cancer cell proliferation, survival, and invasion [82,83]; and pave the way for the guided invasion of tumor cells by generating ECM tracks [84]. Diverse CAF-derived collagen modifying enzymes such as PLOD2 and LOXL2 have been shown to be involved in the modification of ECM stiffness [85]. Therefore, CAF-mediated ECM remodeling and stiffness enhance cancer cell invasion and metastasis. Moreover, the increased expression of N-cadherin on endothelial cells induced by ECM stiffness facilitates the binding of cancer cells to the endothelium and the intravasation into the blood vessels [86]. This, together with the proangiogenic action of some CAF-secreted growth factors, such as vascular endothelial growth factor (VEGF), fibroblast growth factor (FGF), and platelet-derived growth factor (PDGFC), and cytokines, such as CXCL12 (also known as stromal cell-derived factor 1 or SDF-1) and CXCL14 [87], enables tumor growth and cancer cell dissemination. On the other hand, CAFs can enhance the metastatic potential of cancer cells by the induction of EMT, through the paracrine secretion of transforming growth factor-β (TGF-β),which promotes cancer cell invasion [88,89] and the differentiation of CAFs in an autocrine fashion [90], CXCL12 [91], interleukin 6 (IL-6) [92,93], or matrix metalloproteinases (MMPs) [94]. CAF-induced EMT is also shown in experimental settings using conditioned media and co-cultures [95,96,97,98]. In addition, TGF-β together with some chemokines and cytokines such as IL-6 and CXCL9 promote cancer progression by suppressing the antitumor immune response leading to the disruption of T cell function and cancer cell immune evasion [99,100]. Moreover, the interaction mediated by cytokines between CAFs and TAMs, known regulators of critical steps in tumor metastasis [101,102], is another factor contributing to the promotion of invasion and metastasis [103]. 

In addition to the mechanisms above, CAFs can also affect the biology of cancer cells via the release of extracellular vesicles, commonly known as exosomes. Exosomes are vehicles for the transport of proteins and nucleic acids (miRNAs, lncRNAs) to both cancer and stroma cells that can promote cancer cell migration and aggressiveness [104,105,106,107]. Thus, fibroblast-derived exosomes are now considered as positive mediators of cancer progression and stromal remodeling [63] as well as mediators of chemoresistance [108,109,110]. As part of a bidirectional crosstalk, cancer cell-derived exosomes can promote the transformation of CAFs [111]. For example, exosomes secreted by high-metastatic hepatocellular carcinoma cells induce CAF activation in the lungs, facilitating lung metastasis [112]. Tumor-derived exosomes, together with other soluble factors, are transported by the blood to target tissues where they will help to establish the so called “pre-metastatic niche” [113], a permissive environment for the establishment of disseminated tumor cells, where they can lodge and proliferate [114]. A study by Kaplan et al. shows that bone marrow-derived hematopoietic progenitor cells that express vascular endothelial growth factor receptor 1 (VEGFR1) are attracted by deposits of fibronectin secreted by resident fibroblasts just days after tumor implantation, establishing a pre-metastatic niche for the arrival of tumor cells [115]. This work suggests that tumor-secreted factors promote metastatic spread into specific distant organs in an orchestrated way, a process in which fibroblasts seem to be involved. Indeed, other studies support a critical role for fibroblasts in the formation of the metastatic niche [116,117,118,119]. 

Furthermore, CAFs are likely to contribute to the establishment of the pre-metastatic and metastatic niche in a direct fashion, through their mobilization from the primary tumor and recruitment to metastatic sites. The analysis of blood samples from mouse models and cancer patients has revealed the presence of CAFs in circulation either in the company of CTCs, or as individual CAFs and in clusters [56,59]. Moreover, fibroblast progenitor cells are found in the peripheral blood of patients with lung cancer, in the pulmonary veins, which can be recruited into the cancer stroma [120]. CAFs are also detected in ascites fluid from patients with ovarian cancer [121], and fibroblast-like cells in the blood of metastatic prostate cancer patients [122]. These data indicate the existence of circulating fibroblasts contributing to the pool of CAFs found at metastatic sites that could favor metastatic outgrowth of CTCs once disseminated through the bloodstream. In addition, they also suggest that CAFs might have a role in the genesis of CTC clusters.

## 4. Role of CAFs in CTC Cluster Formation

CTC clusters can derive from the primary tumor, probably due to a phenomenon of collective cell migration, or from the intravascular aggregation of single CTCs [25,51]. Contrary to the mesenchymal type of migration of individual carcinoma cells, mediated by the process of EMT, carcinoma cells can also collectively migrate in a coordinated fashion as a group while maintaining stable cell-cell adhesion [123,124]. It is believed that within these groups, cancer cells exhibiting mesenchymal features would act as leaders, accompanied by follower cells retaining an epithelial phenotype [125,126]. Accordingly, CTC clusters found in breast and pancreatic cancer patients can show both epithelial and mesenchymal-like phenotypes [48,127], and it seems that the hybrid EMP phenotype is established at the tumor, before invasion into the circulation [128]. Interestingly, the data support a physical role for CAFs as leading cells at the front of the cancer cell collective migration, suggesting that CAFs may mediate CTC cluster formation.

### 4.1. CAF Contribution through Direct Physical Interactions

Fibroblasts trigger proteolytic and structural modifications of the ECM to create channels that precede invading chains of cancer cells. A study by Gaggioli et al. shows that CAFs lead the invasion of squamous cell carcinoma (SCC) cells by generating ECM tracks along which they move as collective chains. This effect is not observed when SCC cells are incubated with a CAF conditioned medium [84], suggesting the need for a physical interaction between CAFs and cancer cells. In a lung adenocarcinoma mouse model, CAFs facilitate collective tumor cell invasion by leading chains of invading epithelial cancer cells [129]. Immunohistochemical examination of primary tumor and metastasis sections reveals the localization of CAFs at the invasive fronts of the primary tumors, and surrounding collective invading tumor cells at the metastatic site. The same pattern is also observed in human lung adenocarcinoma samples, where heterotypic interactions between CAFs and clusters of cancer cells maintain collective invading chains [129]. Interestingly, this study suggests that CAF recruitment contributes to tumor metastasis by promoting collective invasion of cancer cells and facilitating metastasis to the secondary site. Similarly, in a 3D co-culture system with scirrhous gastric carcinoma cells, CAFs induce the formation of invasive cellular aggregates and locate themselves at the center and the leading front, but only when both cell types were in direct association [130]. Moreover, CAFs can exert pulling physical forces on cancer cells mediated by the adhesion of N-cadherin and E-cadherin at the CAF and cancer cell membranes respectively, providing a cooperative invasion mechanism [131]. The impairment of these heterotypic adhesions limits cancer cell invasion due to a disruption on the guidance ability of CAFs, which fail to lead collective migration. In line with the above observations, a zebrafish embryo model of metastasis shows that disseminated CAFs remain in close association with tumor cells at the metastatic foci formed, and that they promote the metastatic capacity of cells from different cancer types [132]. Although in this occasion the mechanism behind is not studied, the results indicate an interaction between both cellular types maintained even after the intravasation of the cancer cells. Collectively, these studies support that a physical direct interaction between CAFs and cancer cells may mediate the formation of heterotypic clusters.

### 4.2. CAF Contribution through Indirect Mechanisms

CAFs can also guide the migration of cancer cell clusters without the need for a direct interaction. By applying mechanical pulling forces on ECM fibers, CAFs generate gaps in the basement membrane facilitating cancer cell invasion independent of metalloproteinases [133]. These results suggest that CAFs might contribute to the generation of CTC clusters through a mechanism implying a physical interaction between them and the cancer cells through the basement membrane.

Besides, CAFs may be involved in tumor cell cluster formation by promoting in a paracrine fashion the invasion of groups of cancer cells, through the induction of EMP. A recent work in breast cancer shows that CAF-released SDF-1 and TGF-β drive the formation and maintenance of a hybrid population of epithelial/mesenchymal (E/M) cells and highly epithelial (E) cells within clusters of cancer cells [134]. E/M cells are induced by the expression of ZEB1 and lead the collective migration, while E cells, acting as follower cells, preserve cell-cell adhesions that enable cluster formation and show a higher metastatic seeding ability in mouse models [134]. Interestingly, in addition to the presence of hybrid CTC clusters in the blood of mice, this hybrid population also exists in primary human breast carcinomas [134]. Of note, despite the involvement of CAFs in tumor cell cluster formation and higher incidence of lung metastases in mice co-injected with CAFs (than with normal or no fibroblast), in this particular study, CAFs are not found at the lung metastases [134]. This observation may suggest that no physical interaction between CAFs and cancer cells is required, and that CAF-secreted factors induce EMP in a paracrine fashion promoting the formation of homotypic rather than heterotypic CTC clusters. However, this work does not assess the possible presence of CAFs within the clusters in the bloodstream of the mice. 

**Figure 1:**
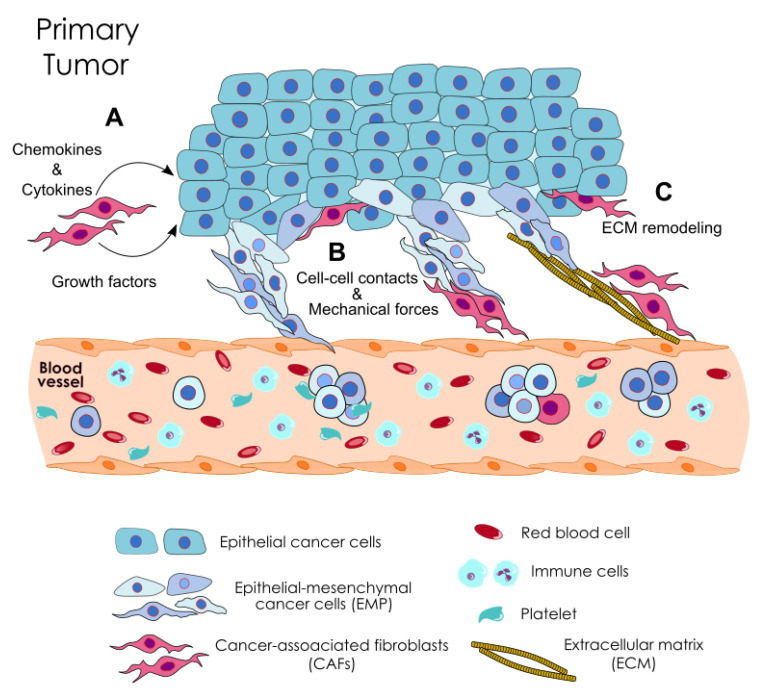
Role of CAFs on the formation of CTC clusters. CAFs can contribute to the formation of CTC clusters by at least three mechanisms. (**A**) CAFs secreted factors can reprogram cancer cells, inducing epithelial/mesenchymal plasticity, which endows an invasive phenotype. (**B**) CAFs exert mechanical forces on cancer cells that direct collective migration through the establishment of heterotypic cell-to-cell interactions. (**C**) CAFs trigger structural modifications of the ECM, by generating ECM tracks or by applying mechanical pulling forces on ECM fibers that favor collective cancer cell invasion.

In agreement with the role of CAFs in the formation of CTC clusters through the induction of EMT, indirect evidence is also shown by a prostate cancer mouse model [94]. CAF co-injection with tumor cells results in the induction of EMT through the secretion of metalloproteinases, and on an enhanced tumor growth and metastasis formation. Histological examination of primary tumors from these mice reveals the presence of several emboli of carcinoma cells inside peripheral venules [94], suggesting the occurrence of collective migration and intravasation of tumor cells. On the other hand, EMT-mediated cancer cell motility by the heterotypic direct interaction between CAFs and cancer cells as well as CAF-facilitated collective cell migration independent of EMT is also observed [97,129]. Collectively, these data support the co-existence of at least two different mechanisms by which CAFs may promote the formation of CTC clusters, (i) a mechanical/physical effect assisting the collective migration of cancer cells, and (ii) a molecular reprograming by which CAFs induce epithelial/mesenchymal plasticity on cancer cell endowing an invasive phenotype (Table 1 and Figure 1).

## 5. Contribution of CAFs to the Metastatic Potential of CTC Clusters 

**Figure 2:**
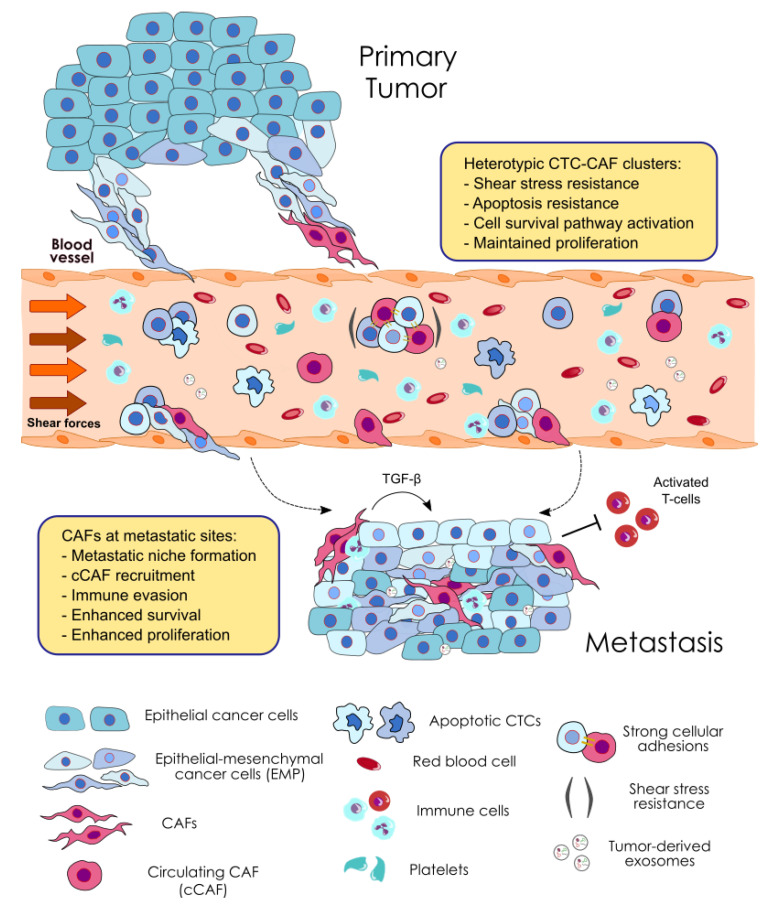
Pro-metastatic effects exerted by CAFs within heterotypic CTC clusters and at the secondary site. In circulation, CAFs enhance the metastatic potential of CTCs by conferring to them shear stress resistance through the establishment of strong cellular adhesions. Moreover, CAFs protect CTCs from apoptosis by activating cell survival pathways and maintaining CTC proliferation. At the metastatic site, CAF-secreted factors and tumor-derived exosomes create an appropriate environment for CTC growth, also supported by CAF-mediated immunosuppression. Additionally, circulating CAFs can be recruited to secondary sites and contribute to the establishment of the metastatic niche.

In addition to the involvement of CAFs in the genesis of CTC clusters, they can also contribute to the metastatic potential of CTCs (Figure 2 and Table 2). As previously described, many studies indicate that CAFs mediate tumor metastasis formation [94,129,132,134]. However, these studies do not investigate the role of CAFs as CTC modulators as part of heterotypic CTC clusters. In this regard, Duda et al. are the first showing that the presence of CAFs in heterotypic CTC clusters facilitates the formation of metastases in a mouse model of lung cancer metastasis [56]. The analysis of the content of the efferent blood from tumor bearing mice demonstrates that CTCs can bring stromal fibroblast from the primary tumor to the metastatic site, as determined by the immunohistochemical characterization of stromal cells. Moreover, stromal cells at distant sites, mainly represented by CAFs, survive and proliferate, providing a survival advantage for the establishment of metastatic foci. Importantly, the depletion of CAFs from the lungs significantly reduces the number of macroscopic metastases observed and extends mice survival [56]. These data indicate that CAFs within CTC clusters promote the formation of lung metastases, although the contribution of other stromal cellular components cannot be ruled out. In addition, this work also shows that fibroblasts enhance the survival of CTCs, providing a growth advantage at distant sites. Heterotypic CTC clusters from the blood of these mice show a lower incidence of apoptotic cells and a significantly higher number of viable cells than single CTCs or homotypic clusters formed by two or three cancer cells [56]. Clinical studies have shown the low incidence of apoptotic CTCs within homotypic CTC clusters compared to single CTCs [31,43,135], therefore, this result suggests that CAFs present in clusters can further enhance the survival of CTCs in circulation. 

In line with their pro-survival effect, CAFs can help cancer cells to survive in circulation by conferring resistance to the fluid shear forces [136], the main cause of CTC death in the circulation [137]. In spheroid co-cultured under high magnitude fluid shear stress (FSS), CAFs induce shear resistance of prostate tumor cells via strong cellular adhesions and soluble factors, which promote stable cell aggregates, and can activate signaling pathways involved in cell survival, invasion and EMT, respectively (Figure 2). Under the conditions tested, the viability and proliferative capability of cancer cells is preserved [136]. Furthermore, recent data present a relationship between shear stress forces and EMT in epithelial tumor cells [138,139], which suggests that EMP observed in CTC clusters can positively influence CTC survival against circulatory forces and metastasis seeding efficiency. This concept is not new to the CTC cluster literature as it has been hypothesized that the cooperation of CTCs within a cluster with EMP features may represent a mechanism of resistance to circulatory stresses [140,141]. Consequently, we could reason that CAF-induced EMP can be an additional mechanism shaping the survival and metastatic competency of CTCs in clusters, although further direct mechanistic evidence is needed. In summary, we can speculate that CAFs may help CTCs to survive in circulation by priming cancer cells at the primary site and within heterotypic CTC clusters. Furthermore, they may contribute to create a suitable environment for CTCs to survive and proliferate at distant sites, probably protected from immune attack due to CAFs-mediated immune evasion [100].

## 6. Combined Analysis of CTCs and CAFs: Expanding on Circulating Biomarkers

Clinical evidence seems to suggest the use of CAFs as a potential biomarker of poor prognosis in some cancer types [59]. Ao et al. show a significant association between the presence of CAFs in the circulation of breast cancer patients and the presence of metastatic disease, with these cells predominantly found clustering with CTCs [59]. They process patient blood samples through a microfilter and enumerate CTCs (defined as cytokeratins (CKs) positive and CD45 negative cells) and CAFs (defined as α-SMA/FAP positive, and CKs and CD45 negative cells) based on immunofluorescent staining. CAFs are further identified by the spindle-like morphology displayed by the captured cells in culture, characteristic of cells of mesenchymal origin. Blood from patients with stage IV metastatic breast cancer shows a much higher frequency of circulating CAFs than blood from stage I patients treated with curative therapy who had no evidence of disease (>5 years of long-term disease-free survival), which goes in line with the significantly higher number of absolute CTC counts in the metastatic group. Of note, the clustering of CTCs and CAFs is exclusively detected in the blood from metastatic patients, suggesting that fibroblasts may actively participate in the dissemination of CTCs and metastasis seeding [59]. However, in a later work the same authors suggest that heterotypic CTC-CAF clusters are also found in the blood of patients with earlier stage breast cancer [142], although at the time of writing these lines these data had not been yet published. In addition, the study also shows that circulating CAFs are detected in peripheral blood from metastatic colorectal and localized prostate cancer patients. Furthermore, in blood samples from four patients (one with metastatic breast cancer, two with metastatic colorectal cancer, and one with localized prostate cancer) circulating CAFs were detected in the absence of CTCs [59]. These findings reinforce the idea that this population of cells may be a standalone indicator of a poorer prognosis, and may also indicate that they can reach secondary sites on their own, where they can help cancer cells grow by establishing a permissive environment. 

In a similar work using the CellSearch^®^ system, circulating fibroblast-like cells, defined as vimentin and DAPI positive, and CKs and CD45 negative cells, are present in the blood of patients with metastatic prostate cancer, while they are not found in patients with localized prostate cancer and healthy individuals [122]. These cells cluster with CTCs (defined as CKs and DAPI positive, and CD45 negative cells), and their presence correlates with the presence of CTCs (≥5 CTCs), a known indicator of poor prognosis in patients with metastatic prostate cancer [143]. It is important to notice that the combination of markers used in this study to identify circulating fibroblasts differ from the combination in the study by Ao et al., who defined them based on the expression of α-SMA and FAP, probably the most widely accepted markers for defining a CAF. Of note, the expression of the mesenchymal marker vimentin is not restricted to fibroblast, and other cell populations, including EMT CTCs, can express it. Therefore, further characterization (e.g., genomic analyses) will be needed to determine whether these cells are truly fibroblasts and not tumor cells. Nevertheless, both studies suggest that CAFs may represent an independent prognostic factor for metastatic breast and prostate cancer patients, and that the analysis of circulating CAFs could be used in combination with the enumeration of CTCs as prognostic biomarkers. Although these observations need validation in larger patient cohorts, taken in combination with preclinical data available, they indicate that CAFs can shape the biology of tumor cells, and in particular of CTCs. This evidence grants further investigation on the role of heterotypic CTC clusters in metastasis regarding the biological interactions between CAFs and CTCs and the clinical implications.

## 7. Therapeutic Interventions Involving CAF Targeting

Research efforts are being driven towards the development of pharmacological interventions that might limit the participation of CTC clusters in the process of metastasis. This goal could be achieved in two fashions: (i) preventing the formation of tumor cell clusters at tumor sites and their release into the circulation, and (ii) targeting/disrupting the clusters while transitioning in circulation to limit their metastatic action.

With regard to the first approach, there is a need for the identification of those factors that promote CTC cluster formation, whether they are cancer-cell intrinsic, derived from the tumor microenvironment or both, as well as the molecules involved. Studies have shown that the genetic targeting of plakoglobin and keratin 14, both involved in cell-cell junctions, reduce CTC cluster formation and metastasis incidence [25,26]. In order to prevent CTC clusters from abandoning the tumor, therapeutic interventions aimed to target CAFs may be useful in limiting tumor spread. A few therapeutic strategies directed to interfere with CAFs are currently being evaluated in clinical trials, most of them aimed to eliminate or to reprogram them to a “normal” fibroblast state. Two examples are FAP targeting strategies or the activation of the Vitamin D receptor (therapeutic strategies are summarized in detail in [64,69]). However, to inhibit the formation of CTC clusters mediated by CAFs, a deeper knowledge about the heterogeneity of the fibroblasts, and how it affects the interaction with cancer cells (both at the tumor site and in circulation) is needed. In addition, a deeper insight into the molecular mechanisms involved in CAF-mediated cluster formation is required, whether it is mediated by cell-cell interactions between CAFs and cancer cells or by the invasion-promoting remodeling of the ECM (through CAF applied mechanical forces or CAF-secreted factors). In this regard, an interesting strategy would be to develop molecules able to disrupt direct contacts between CAFs and tumor cells. Preliminary data in this sense are available showing that the Src inhibitor dasatinib blocks the physical interaction between CAFs and cancer cells, reducing the incidence of peritoneal metastases in a mouse model of scirrhous gastric carcinoma [144]. Another strategy would be to interfere with the crosstalk between CAFs and cancer cells, in particular with those CAF-derived signals that promote tumor cell clustering and invasion. However, it seems necessary to first identify markers for those subpopulations of CAFs that promote the collective migration and invasion of tumor cells, whatever the mechanism. Nevertheless, the functional and genetic heterogeneity of CAFs represents an important challenge to overcome, as therapies specifically targeting these subpopulations and/or the reversibility among them will be required to control the dissemination of cancer cell clusters from the primary tumor.

Related to the second approach, few preclinical studies are showing the validity of disrupting CTC clusters as an anti-metastatic approach. Example for this are the studies evaluating the Focal Adhesion Kinase (FAK) inhibitor and the Na+/K+-ATPase inhibitor ouabaïn, which have shown promising results in preclinical models [50,145]. In this regard, since CAFs can be found associated with CTCs, it would be interesting to find out if they play a role in maintaining CTC cluster integrity. In support of this, the work by Ortiz-Otero et al. suggests that CAFs and prostate cancer cells form stable cell aggregates mediated by strong cellular adhesion which protect cancer cells from FSS, enhancing CTC survival and proliferation [136]. Therefore, this evidence might suggest that agents targeting circulating CAFs could potentially have a beneficial effect by dissociating CTC clusters and thus, limiting metastasis. On the other hand, it is suggested that cluster “compactness” may predict patient treatment response in different cancer types, with very tight clusters corresponding to patients with advanced disease and lose ones to patients who had no measurable disease [146]. Therefore, we could speculate that if CAFs are involved in CTC cluster integrity, in addition to their role as mediators of resistance to therapy (including mediated immunosuppression) [64], targeting them could be a potential therapeutic strategy aimed to make CTC cluster more vulnerable and accessible to therapy. Of course, such hypothesis should be first evaluated in preclinical models.

## 8. Outlook and Conclusions

The literature on CTC clusters supports their prominent role in cancer dissemination. This is a field of growing interest in cancer metastasis research, aided by the technological advancement for the isolation and molecular characterization of these cells. Although most of available data in this regard are in homotypic CTC clusters, it is becoming clearer that the interactions between other stromal milieu cellular components and the CTCs within heterotypic clusters is an important determinant of CTC survival and metastatic competency [147]. In this sense, the evidence gathered on the role of CAFs supports a pro-metastatic effect enhancing the capacity of CTCs to seed distant lesions, although the literature is still limited. 

Many questions about the biology of CTC clusters, and in particular about the heterotypic CTC-CAF clusters, need to be investigated in order to be able to understand the real metastatic potential of these cellular aggregates and their contribution to metastasis. For instance, at the level of the primary site, we need to clarify what subpopulation/s of CAFs is/are more likely to interact and/or co-invade with tumor cells promoting CTC cluster formation. We also need to learn whether CAFs selectively interact with cancer cells bearing specific mutations (clones), and therefore, if CAFs are a contributing factor to the polyclonality of CTC clusters and metastatic successes. In addition, and at the circulatory level, we need to shed light on what are those CAF-derived signals that enhance CTC-survival and metastasis efficiency, as well as to determine the functional differences (immune evasion, therapy resistance, etc.) between homotypic CTC cluster and heterotypic CTC-CAF clusters. Moreover, and very importantly, we need to understand how frequent these heterotypic clusters are in the blood of patients with cancers at different stages of development to evaluate their utility as biomarkers for the detection of metastasis, monitoring of disease progression, and patient prognosis in response to therapy.

Altogether, the evidence on the role of CTC clusters in metastasis and the contribution of non-cancer cells in the process make this a very exciting field of research. The current available technologies for the isolation and characterization of CTCs, NGS and single cell analyses, together with advanced experimental models will allow answering some of the above questions. However, in order to do so, more insight into the heterogeneity and plasticity of CAFs along with the identification of specific markers is required. This, should also be accompanied by the validation of the origin of the circulating CAFs, to determine whether they come from the tumor (when they are found in the absence of CTCs), as well as to verify that they are true CAFs and not tumor cells. Similarly, a deeper understanding of the heterogeneity of the CTCs within a cluster is needed to identify possible weaknesses enabling the development of therapeutic interventions aimed to delay and even limit the onset of metastasis.

## Figures and Tables

**Table 1:** Direct or indirect evidence of the contribution of CAFs to CTC cluster formation or CTC cluster metastatic potential.

**CAF Contribution through Direct Physical Interactions Driving Collective Cancer Cell Invasion**				
**Tumor specimen and assay**	**CAF origin**	**Markers used**	**Findings**	**References**
SCC cells cocultures	Isolated from H&N and vulval SCC patient samples	Vimentin	CAFs lead collectively invading chains of cancer cells by the physical remodeling of the matrix	[84]
LAC mouse model, spheroids and patient samples	Mouse and human LAC	α-SMA, FSP1 and vimentin	CAFs establish heterotypic interactions with tumor cells and lead collective invasion and metastasis, mediated by vimentin	[129]
SGC cells 3D cocultures	Established from SGC patient samples	α-SMA, FSP1and vimentin	CAFs locate at the center and the leading front promoting invasive cancer cell aggregates	[130]
Human vulval SCC and LAC cells cocultures	Human vulval SCC and LAC	α-SMA	Mechanically active heterophilic N-cadherin/E-cadherin adhesion between CAFs and cancer cells enables cooperative tumor invasion	[131]
Human HCC, SCC, PDAC, and LC and BC cell lines injected in zebrafish	Isolated from CRC patient tumor and mouse fibrosarcoma; human PC	α-SMA, FSP1, desmin, and PDGFRα	CAFs promote the metastatic capacity of tumor cells in zebrafish, and remain in tight association with cancer cells in the circulation	[132]
**CAF contribution through indirect mechanisms (i.e., paracrine effect)**				
**Tumor specimen and assay**	**CAF origin**	**Markers used**	**Findings**	**References**
Human CRC cells and patient samples	Isolated from human colon tumors	α-SMA and FAP	CAFs generate gaps in the basement membrane facilitating cancer cell clusters invasion independent of MMPs	[133]
BC cell lines cocultures and mouse xenografts models, and BC tissues samples	Isolation of immortalized human mammary fibroblasts from MCF-7-ras tumor bearing mice	α-SMA and tenascin-C	Paracrine secretion of SDF-1 and TGF-β by CAFs drives cancer cell clusters formation via EMP. Hybrid E/M clusters found in patients’ tissue samples	[134]
PC cells conditioned media and mouse xenografts	Isolated from human PC tumor samples	α-SMA and FAP	CAF-secreted MMPs induce EMT and enhance tumor growth and metastasis. Emboli of cancer cells found inside peripheral venules of mice tumors	[94]

Abbreviations: α-SMA, alpha-smooth muscle actin; BC, breast cancer; CRC, colorectal cancer; E/M, epithelial-mesenchymal; EMP, epithelial–mesenchymal plasticity; EMT, epithelial-to-mesenchymal transition; FAP: Fibroblast activation protein; FSP1, fibroblast specific protein-1; HCC, hepatocellular carcinoma; H&N, head and neck; LC, lung cancer; LAC, lung adenocarcinoma; MMPs, matrix metalloproteinases; PC, prostate cancer; PDAC, pancreatic ductal adenocarcinoma; PDGFRα, platelet-derived growth factor receptor-α; SGC, scirrhous gastric carcinoma; SCC, squamous cell carcinoma.

**Table 2:** Evidence of the contribution of CAFs to the metastatic potential of CTC clusters.

Tumor Specimen and Assay	CAF Origin	Markers Used	Findings	References
BC, CRC, and PC patient´s blood samples	Circulating fibroblasts from human BC, CRC and PC	α-SMA and FAP	Presence of heterotypic CTC-CAF clusters in the blood of metastatic BC patients and circulating CAFs in the blood of CRC and PC patients	[59]
PC patient´s blood samples	Human PC	Vimentin	Circulating fibroblast-like cells found clustering with CTCs in the blood of patients with metastatic PC	[122]
LLC mouse model	Mouse LLC model and isolated from human BC tumor samples	α-SMA and FSP1	CAFs in heterotypic CTC clusters facilitates the formation of metastases in a mouse model of LC metastasis	[56]
PC cell lines 3D cocultures under FSS assays	hTERT-immortalized human PC fibroblasts	α-SMA, FAP and FSP1	CAFs promote the survival of cancer cells in circulation by conferring resistance to the FSS	[136]

Abbreviations: α-SMA, alpha-smooth muscle actin; BC, breast cancer; CRC, colorectal cancer; FAP, fibroblast activation protein; FSS, fluid shear stress; FSP1, fibroblast specific protein-1; LLC, lewis lung carcinoma; PC, prostate cancer.

## Abbreviations

α-SMA	α- smooth muscle actin
BM-MSCs	Bone marrow-derived mesenchymal stem cells
CAF	Cancer-associated fibroblast
CK	cytokeratin
CTC	Circulating Tumor Cell
CXCL9	C-X-C Motif Chemokine Ligand 9
CXCL12	C-X-C Motif Chemokine Ligand 12
CXCL12	C-X-C Motif Chemokine Ligand 14
DAPI	4′,6-diamidino-2-phenylindole
ECM	Extracellular matrix
EMP	Epithelial–mesenchymal plasticity
EMT	Epithelial-to-mesenchymal transition
EV	Extracellular vesicle
FAK	Focal Adhesion Kinase
FAP	Fibroblast activation protein
FGF	Fibroblast growth factor
FSP	Fibroblast specific protein
IL-6	Interleukin 6
LN	lymph nodes
LncRNA	Long non-coding RNAs
miRNA	microRNA
MMP	Matrix metalloproteinases
NGS	Next generation sequencing
PDGFC	Platelet-derived growth factor
PDGFRα	platelet-derived growth factor receptor-α
PDGFRβ	Platelet-derived growth factor receptor-β
PMN-MDSCs	Polymorphonuclear-myeloid-derived suppressor cells
SDF-1	Stromal cell-derived factor 1
SRC	SRC Proto-Oncogene, Non-Receptor Tyrosine Kinase
TAMs	Tumor-associated macrophages
TGF-β	Transforming growth factor-β
TME	Tumor microenvironment
VEGF	vascular endothelial growth factor
VEGFR1	Vascular endothelial growth factor receptor 1
WBC	White blood cell
